# Extrahepatic Biliary Papillomatosis Occurring After Removal of a Dysplastic Gall Bladder

**DOI:** 10.1155/1993/90597

**Published:** 1993

**Authors:** P. M. Sagar, M. Omar, J. Macrie

**Affiliations:** Department of Surgery Scarborough Hospital Scalby Road N. Yorkshire Scarborough Y012 6QL United Kingdom

## Abstract

A case is presented of a woman who developed obstructive jaundice secondary to dysplastic mucinous papillomatosis two years after she had undergone cholecystectomy and exploration of the common bile duct. The gall bladder was dysplastic. It is suggested that the dysplastic glands removed from the common bile duct at the second operation either represented seedlings from dysplastic areas of the gall bladder or were a manifestation of dysplastic field change.


*HPB Surgery*, 1993, Vol. 6, pp. 219–221
Reprints available directly from the publisher
Photocopying permitted by license only

© 1993 Harwood Academic Publishers GmbH
Printed in the United States of America

### CASE REPORT

### EXTRAHEPATIC BILIARY PAPILLOMATOSIS OCCURRING AFTER REMOVAL OF A DYSPLASTIC GALL BLADDER

P.M. SAGAR, M. OMAR and J. MACRIE
*Department of Surgery, Scarborough Hospital, Scalby Road, Scarborough, N. Yorkshire, YO12 6QL, England*

*(Received 13 August 1991)*

A case is presented of a woman who developed obstructive jaundice secondary to dysplastic mucinous papillomatosis two years after she had undergone cholecystectomy and exploration of the common bile duct. The gall bladder was dysplastic. It is suggested that the dysplastic glands removed from the common bile duct at the second operation either represented seedlings from dysplastic areas of the gall bladder or were a manifestation of dysplastic field change.

KEY WORDS: Dysplasia, obstructive jaundice, ERCP

### INTRODUCTION

Extrahepatic biliary papillomatosis is a rare condition. It usually presents with obstructive jaundice or recurrent attacks of ascending cholangitis. The aetiology is obscure but a case is presented here in which the condition occurred after removal of a dysplastic gall bladder. This case raises the possibility that biliary papillomatosis may, in some patients, be a manifestation of the seeding of the biliary tree with neoplastic cells from the gall bladder.

### *Case Report*

A 57 year old woman underwent elective cholecystectomy and exploration of the common bile duct for cholelithiasis and choledocholithiasis. Choledochoscopy was used to ensure complete clearance of the common bile duct. A T-tube was left *in situ* and a subsequent T-tube cholangiogram revealed no filling defects. Unfortunately, removal of the T-tube was difficult and the tube snapped. A further laparotomy was needed to retrieve it. Her subsequent postoperative recovery was uneventful. Histological examination of the gall bladder revealed chronic cholecystitis with several areas of dysplastic change within the epithelium.

Two years later, the patient represented with painless obstructive jaundice. ERCP demonstrated a number of filling defects in the lower end of the common

Address correspondence to: J. MacFie, as above address bile duct (Figure 1). At laparotomy, a transduodenal approach to the common bile duct was chosen to avoid reopening the common bile duct. A sphincteroplasty was carried out and a biliary balloon catheter passed. This allowed the extraction of a solitary stone and an odd grape like mass. Choledochoscopy confirmed complete removal of the mass with clear visualisation of the hepatic radicals of the bile duct. Histological examination of the mass showed a number of dysplastic glands with papillary formation and mucin production — mucinous papillomatosis. No invasion was apparent. The patient's jaundice resolved and she remains well 12 months later.

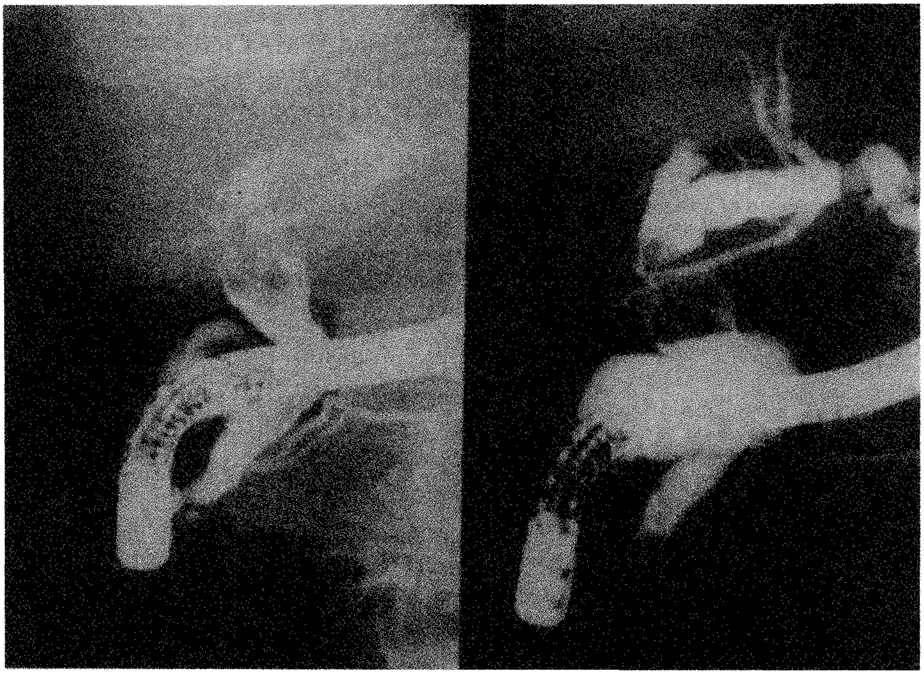

**Figure 1** ERCP which demonstrates multiple filling defects in the lower common bile duct.

### DISCUSSION

Multiple papillomatosis of the biliary tree was first described by Caroli in 1959[1,2]. The common bile duct is the most common site[3]. It may cause intermittent obstructive jaundice as a result of either the papillary mass itself or its viscous mucous secretions[4]. Rarely, it may cause haematobilia[5]. Choledocholithiasis in association with biliary papillomatosis has not been reported previously[5]. Liver failure may supervene as a result of repeated attacks of ascending cholangitis or biliary cirrhosis secondary to unrelieved obstruction.

ERCP facilitates diagnosis and, with the benefit of hindsight, it is possible that this patient could have been successfully treated by endoscopic extraction of the grape-like mass. It would not have been possible, however, to assess fully the extent of clearance of the common bile duct. The filling defects seen on ERCP may be mistaken for air bubbles or blood clots.

Limited surgical procedures are usually used if the lesion is localised. Curettage with a drainage procedure such as sphincteroplasty carries a low morbidity but recurrence may be high[5,6]. The presence of more extensive disease with intrahepatic involvement is an indication for more radical resection such as lobectomy. Nevertheless, even major resection is associated with a high rate of recurrence. Chemotherapy has not produced consistent results[5].

It is presumed that the extrahepatic papillomatosis in this case was related to the dysplastic gall bladder rather than the transiently retained T-tube. Although the latex material used in T-tubes is, by necessity, irritant, the tube was *in situ* only a few days longer than would normally be the case. It is more likely that either the dysplastic papillary cells had arisen from the dysplastic epithelium of the gall bladder or that they represent a field change. If the latter is the case, however, then recurrence seems probable.

### *References*

1. Caroli, J. (1959) Papillomas and papillomatoses of the common bile duct. *Rev. Medicoch, Mal Foie*, **34**, 191–230
2. Caroli, J. (1973) Disease of the intrahepatic biliary tree. *Clin. Gastroenterol.*, **2**, 147–161
3. Dowdy, G.S., Olin, W.G., Shelton, E.L. and Waldron, G.W. (1962) Benign tumours of the extrahepatic bile ducts. *Arch. Surg.*, **85**, 165–175
4. Madden, J.J. and Smith, G.W. (1974) Multiple biliary papillomatosis. *Cancer*, **34**, 1316–1320
5. Gouma, D.J., Singh Mutum, S., Benjamin, I.S. and Blumgart, L.H. (1984) Intrahepatic biliary papillomatosis. *Br. J. Surg.*, **71**, 72–74
6. Padfield, C.J., Amsell, I.D. and Furness, P.N. (1988) Mucinous biliary papillomatosis: a tumour in need of wider recognition. *Histopathology*, **13**, 687–694

*(Accepted by S. Bengmark 18 December 1991)*

